# Supplementary material for: Essential role of lattice oxygen in hydrogen sensing reaction
Source: Nat Commun. 2024 Apr 8;15:2998. doi: 10.1038/s41467-024-47078-x (PMC11001979; doi:10.1038/s41467-024-47078-x)
Supplement: Supplementary file 1 — Supplementary Information [file 41467_2024_47078_MOESM1_ESM.pdf]

Essential role of lattice oxygen in hydrogen sensing reaction

Jiayu Li,[1] Wenzhe Si,[2] Lei Shi,[1] Ruiqin Gao[3,*], Qiuju Li[4,*], Wei An,[1] Zicheng Zhao,[1] Lu Zhang,[1] Ni Bai,[5] Xiaoxin Zou[1] and Guo-Dong Li[1,*]

[1] State Key Laboratory of Inorganic Synthesis and Preparative Chemistry, College of Chemistry, Jilin University, Changchun 130012, China

[2] School of Environment, Tsinghua University, Beijing 100084, P. R. China

[3] School of Biological and Chemical Engineering, NingboTech University, No.1 South Qianhu Road, Ningbo 315100, P. R. China

[4] Department of Chemistry, College of Basic Medicine, Third Military Medical University (Army Medical University), Chongqing 400038, P. R. China.

[5] School of Mechanical and Metallurgical Engineering, Jiangsu University of Science and Technology, Zhangjiagang 215600, P. R. China

* E-mail: gaorq@nbt.edu.cn; liqiuju93@tmmu.edu.cn; lgd@jlu.edu.cn

Keywords: hydrogen detection, gas sensor, energy level engineering, gas-sensing mechanism

Computational details of the depth of lattice-oxygen released from the SGO

In order to estimate the thickness of lattice-oxygen released from the SGO (x in Supplementary Figure 28), we assume that 1) the particle is regarded as a regular sphere, and the diameter of this sphere is set as 5.6 nm according to the size distribution diagram (Supplementary Figure 3), 2) the O atom are assumed distributed uniformly in the particle. Then, the percent of O escape can be corresponding to the ratio between the O escape volume and the whole particle volume, which is described as equation (S1):

$$\text{Oxygen rati}o_{released} = \frac{4}{3} \times \Pi r_1^2 - \frac{4}{3} \times \Pi r_2^2 \qquad \text{(S1)}$$

Considering the experimental results that the escaping percent of O% is about 1.063%, and the value of R is half of diameter, which is 2.8 nm, the value of x can be obtained from the above equation x = 0.045 nm = 0.45 Å.

Based on the Sn-O bond length of approximately 0.2 nm, our findings indicate that only the surface lattice oxygen becomes partially revealed, at an operating temperature of 220°C.

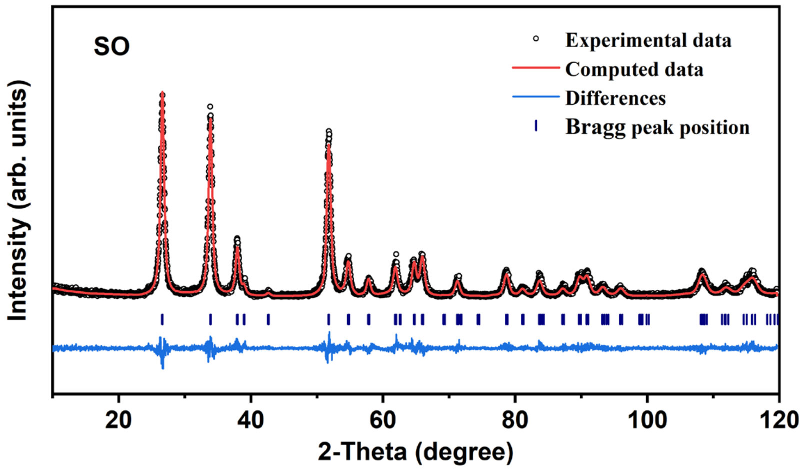

Supplementary Figure 1. XRD pattern with a refinement plot of SnO_2. Source data are provided as a Source Data file.

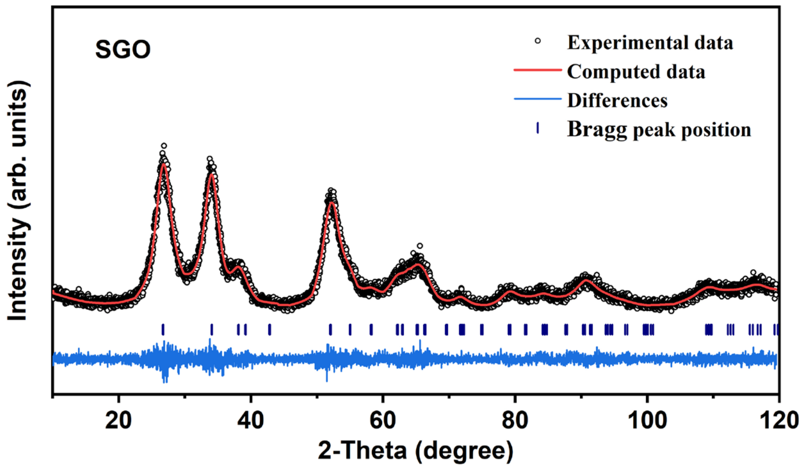

Supplementary Figure 2. XRD pattern with a refinement plot of SGO. Source data are provided as a Source Data file.

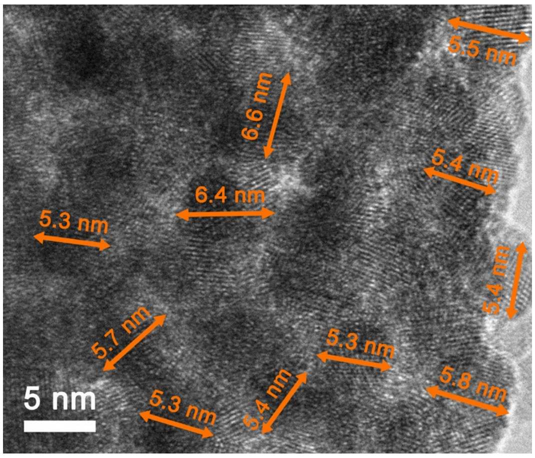

Supplementary Figure 3. TEM image of SGO. Source data are provided as a Source Data file.

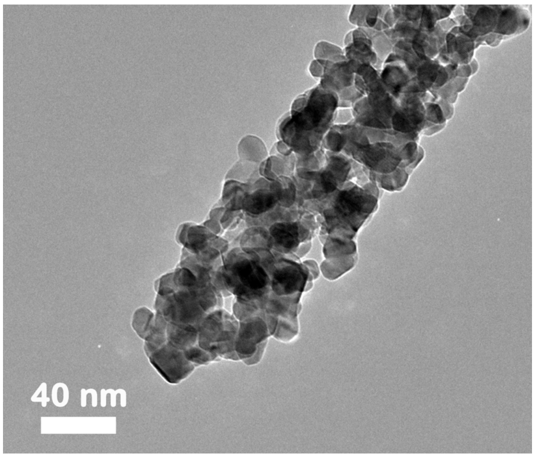

Supplementary Figure 4. TEM image of SO. Source data are provided as a Source Data file.

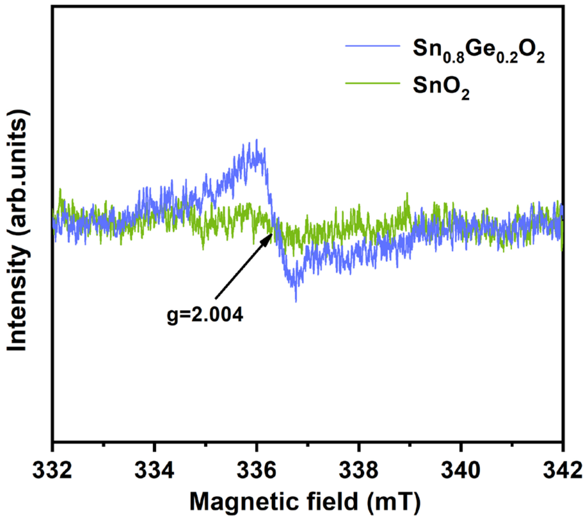

Supplementary Figure 5. EPR spectra of SGO and SO. Source data are provided as a Source Data file.

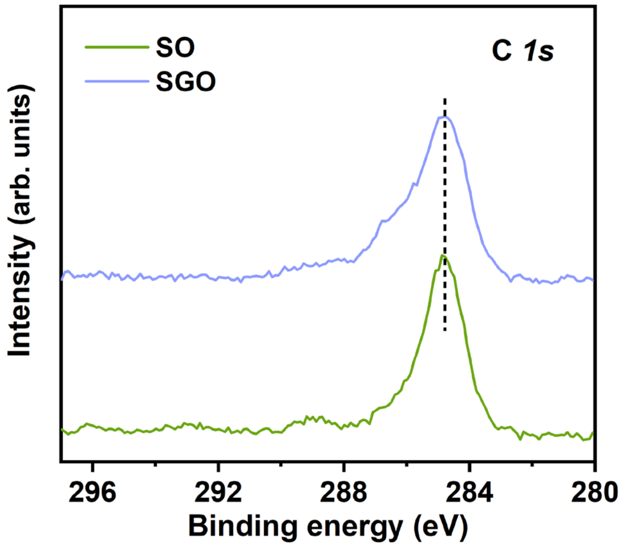

Supplementary Figure 6. The C *1s* XPS spectrums of SGO and SO. Source data are provided as a Source Data file.

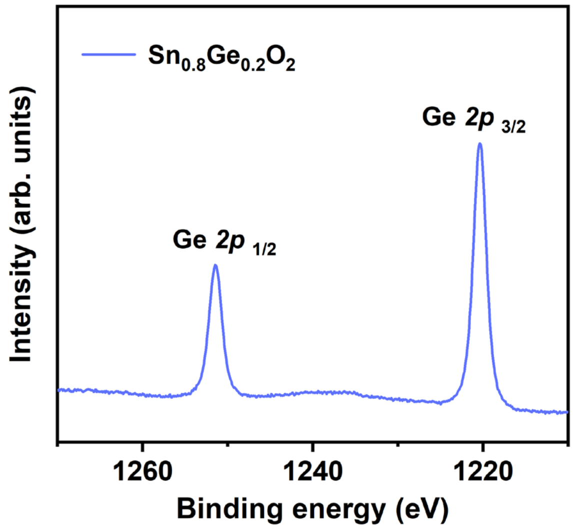

Supplementary Figure 7. The Ge *2p* XPS spectrums of SGO. Source data are provided as a Source Data file.

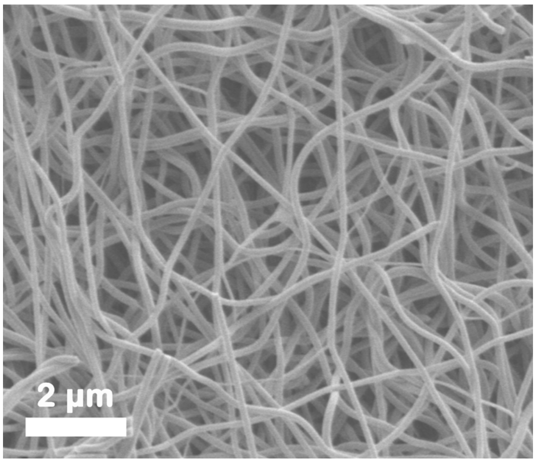

Supplementary Figure 8. SEM image of SGO. Source data are provided as a Source Data file.

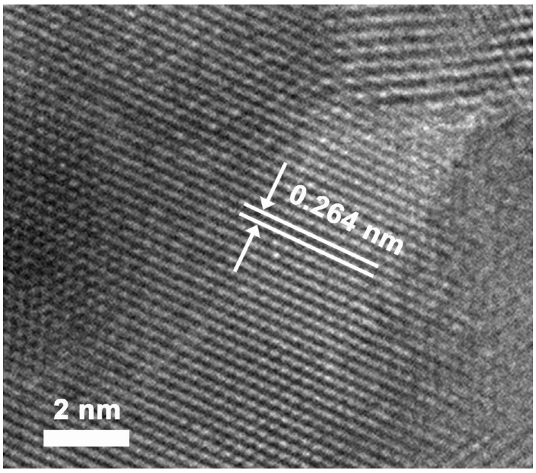

Supplementary Figure 9. HRTEM image of SO. Source data are provided as a Source Data file.

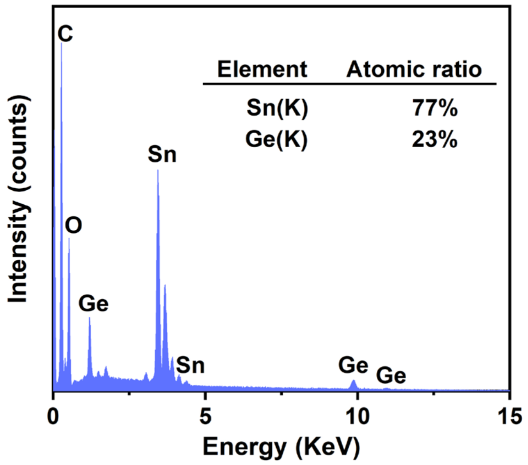

Supplementary Figure 10. The EDS spectrum of SGO. Source data are provided as a Source Data file.

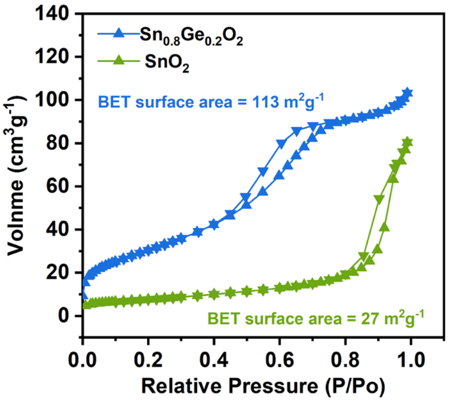

Supplementary Figure 11. N_2 adsorption-desorption isotherms of SGO and SnO_2. Source data are provided as a Source Data file.

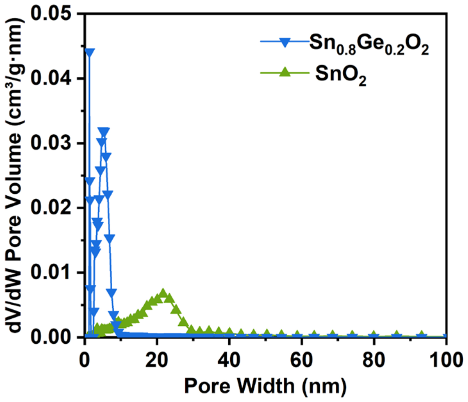

Supplementary Figure 12. Pore size distribution of SGO and SO. Source data are provided as a Source Data file.

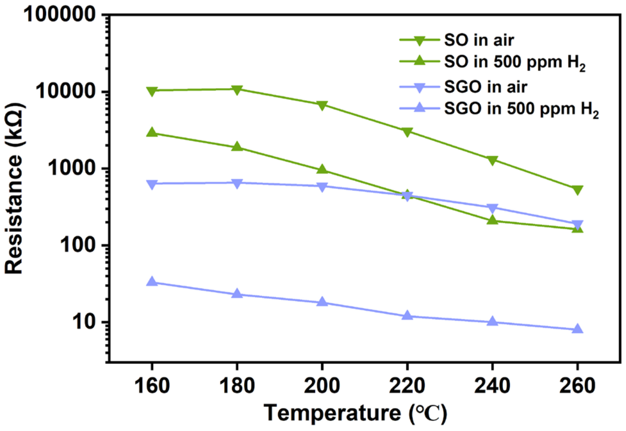

Supplementary Figure 13. The resistance of SGO and SO before and after exposure to H_2. Source data are provided as a Source Data file.

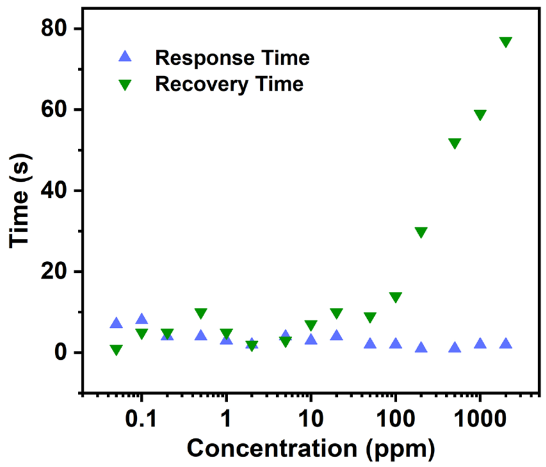

Supplementary Figure 14. The concentration-dependent response time and recovery time of the SGO-based sensor. Source data are provided as a Source Data file.

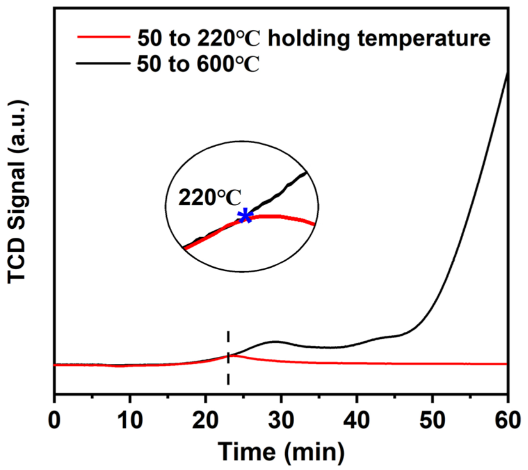

Supplementary Figure 15. H_2-TPR profiles of SGO, red line indicates a heating process where the temperature is gradually increased to 220°C at a rate of 10°C/min, and then maintained at 220°C. On the other hand, the black line represents a heating process where the temperature is directly raised to 600°C at a rate of 10°C/min. Source data are provided as a Source Data file.

To further investigate the LOM mechanism, we performed H_2-TPR analysis on SGO using a 10%H_2/90%Ar mixture. Supplementary Figure 15 illustrates the TPR signal of SGO after direct heating to 600°C and after heating to 220°C and maintaining a constant temperature of 220°C. For SGO heated directly to 600°C, the hydrogen reduction peak emerges at approximately 200°C and gradually intensifies with increasing temperature, indicating that SGO is able to continue to react with hydrogen. However, when SGO is heated to 220°C and held at a constant temperature, the hydrogen reduction peak first rises and then gradually diminishes and eventually disappears. We hypothesize that although surface lattice oxygen can convert to adsorbed oxygen under heating

conditions, this conversion is limited even in an inert atmosphere. Therefore, the saturation observed in the diagram after the reaction can be attributed to the conversion of lattice oxygen into adsorbed oxygen, which participates in the hydrogen reaction, while the deep lattice oxygen remains uninvolved. Consequently, the graph reaches a saturation point after a certain duration. In addition, the viewpoint that deep lattice oxygen does not participate in the reaction is also supported by relevant references (10.1103/PhysRevMaterials.2.054604).

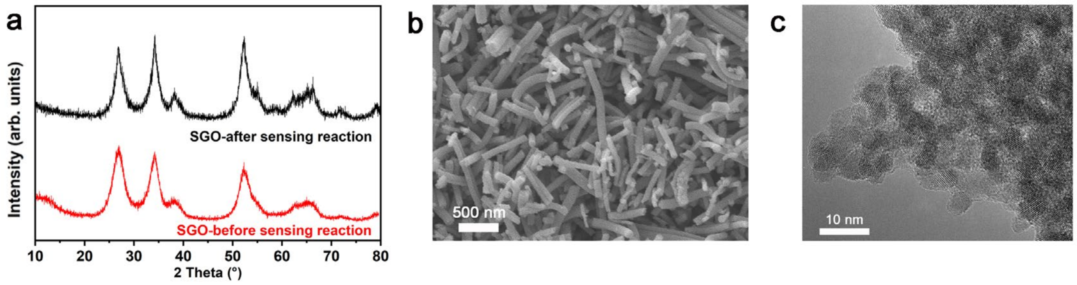

Supplementary Figure 16. (a) XRD patterns of SGO before sensing reaction and after sensing reaction. (b) SEM image of SGO after sensing reaction. (c) HRTEM image of SGO after sensing reaction. Source data are provided as a Source Data file.

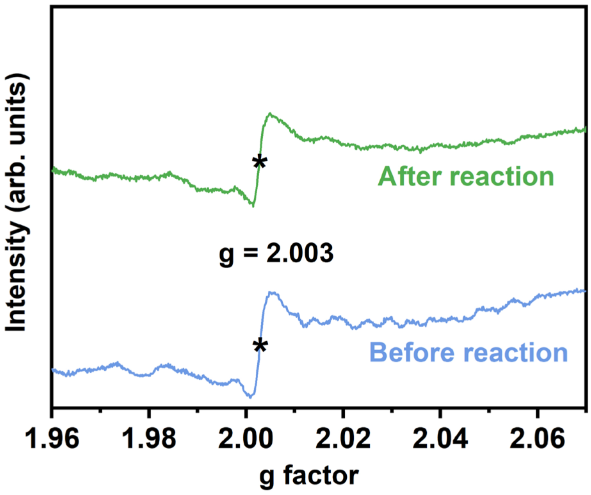

Supplementary Figure 17. EPR spectra of SGO before and after the gas sensing reaction. Source data are provided as a Source Data file.

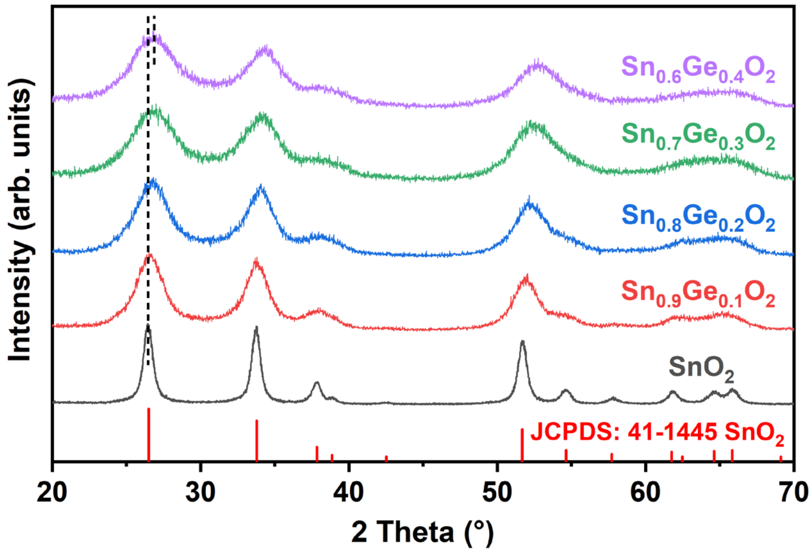

Supplementary Figure 18. XRD patterns of $Sn_{(1-x)}Ge_xO_2$ (x= 0, 0.1, 0.2, 0.3, 0.4). Source data are provided as a Source Data file.

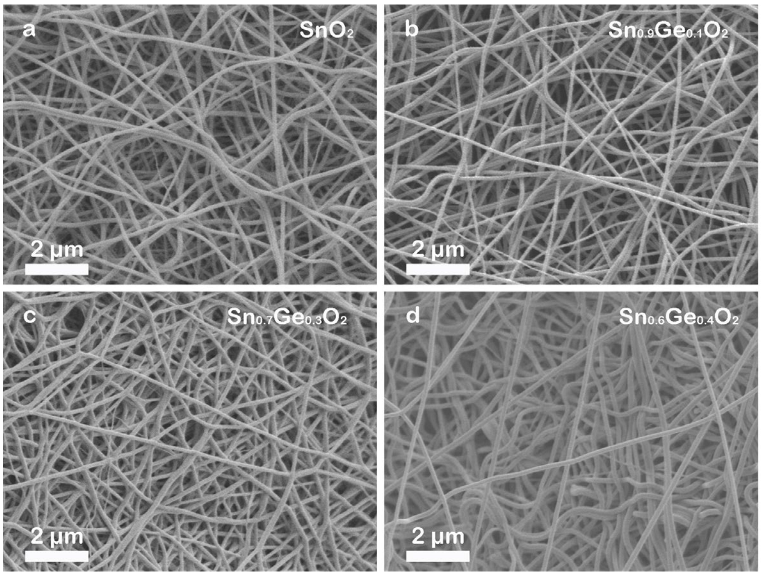

Supplementary Figure 19. SEM images of $Sn_{(1-x)}Ge_xO_2$ (x= 0, 0.1, 0.3, 0.4). Source data are provided as a Source Data file.

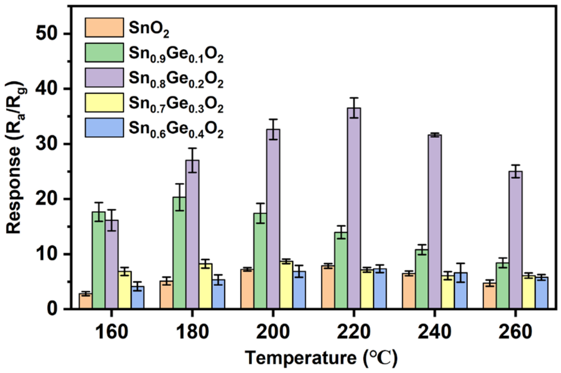

Supplementary Figure 20. Responses of sensors based on $Sn_{(1-x)}Ge_xO_2$ (x= 0, 0.1, 0.2, 0.3, 0.4) to 500 ppm H_2 at 160-260 °C. Source data are provided as a Source Data file.

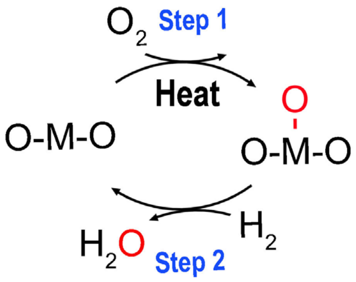

Supplementary Figure 21. Schematic diagram of the mechanism of the conventional gas-sensitive reaction.

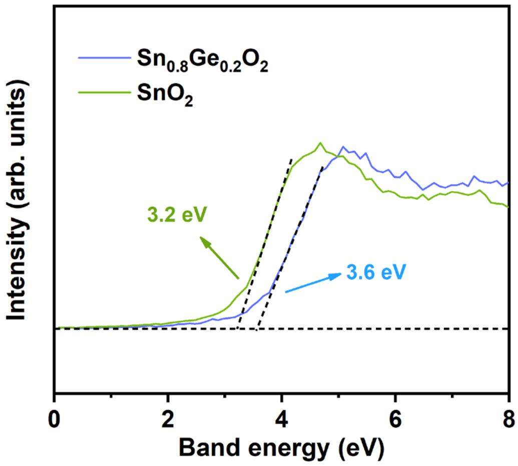

Supplementary Figure 22. The valence band of $Sn_{0.8}Ge_{0.2}O_2$ and SnO_2 were measured by XPS. Source data are provided as a Source Data file.

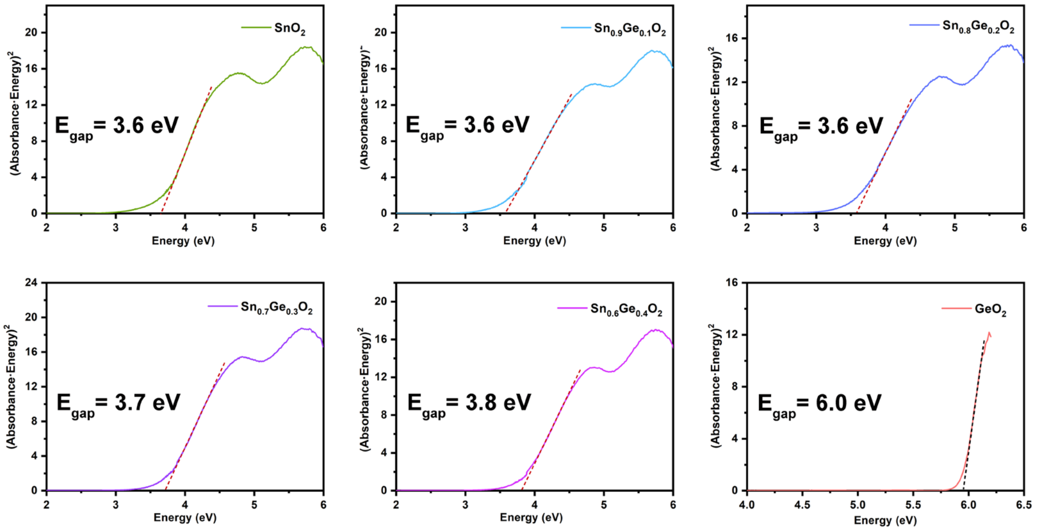

Supplementary Figure 23. Plot of (absorbance·energy)^2 against energy obtained from the data UV/Vis diffuse reflectance spectra of SnO_2, $Sn_xGe_{(1-x)}O_2$ and GeO_2 (x=0.9-0.6). Source data are provided as a Source Data file.

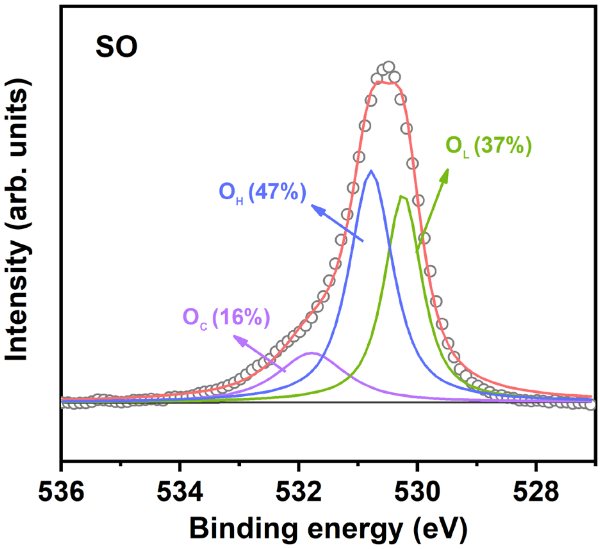

Supplementary Figure 24. The O *1s* XPS spectrum of SO. Source data are provided as a Source Data file.

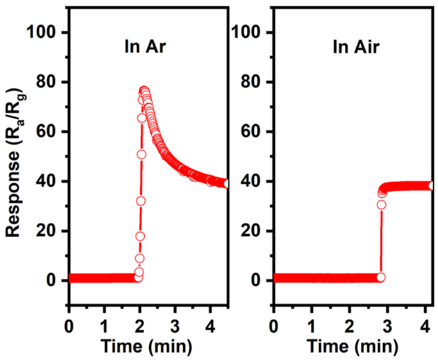

Supplementary Figure 25. Response of sensor based on SGO to 500 ppm H_2 in Ar and air, respectively. Source data are provided as a Source Data file.

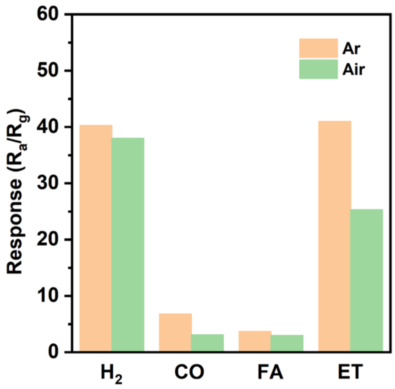

Supplementary Figure 26. The response values of SGO to H_2, CO, formaldehyde (FA), ethanol (ET) under air and argon, respectively. Source data are provided as a Source Data file.

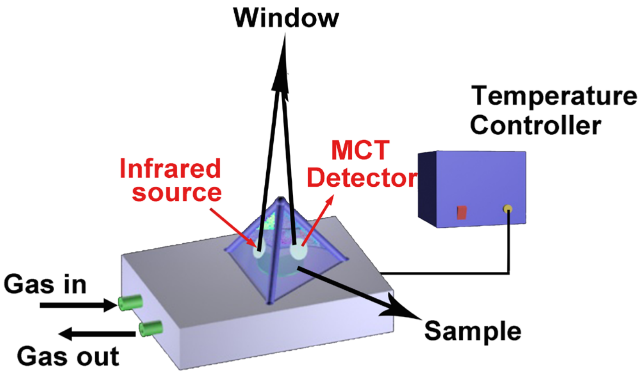

Supplementary Figure 27. The gas-sensing reaction system of in situ IR signal recording.

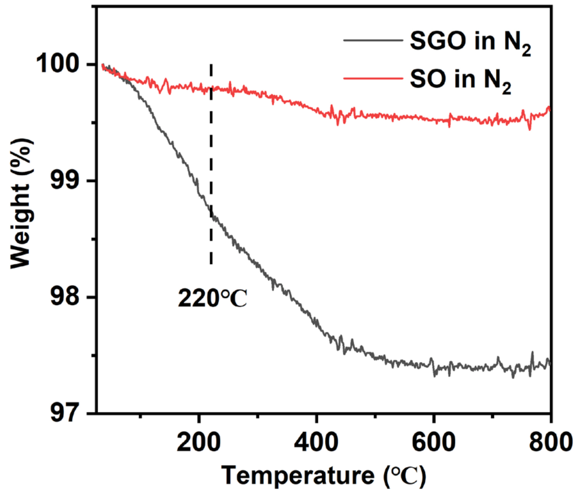

Supplementary Figure 28. TGA plots of SGO and SO in nitrogen. Source data are provided as a Source Data file.

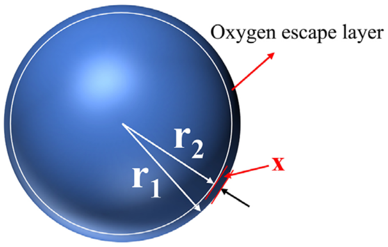

$$\text{Oxygen ratio}_{\text{mass}}=2\times M_O/(0.2\times M_{Ge}+0.8\times M_{Sn}+2\times M_O)\times 100\%=22.61\%$$

$$\text{Oxygen ratio}_{\text{released}}=1.063\%/22.61\%=4.7\%$$

$$V_{\text{particle}}=4/3\times\pi r_1^3$$

$$V_{\text{Oxygen escape layer}}=4/3\times\pi r_1^3-4/3\times\pi r_2^3$$

$$x=r_1-r_2$$

Supplementary Figure 29. The geometry simulation model for SGO particle.

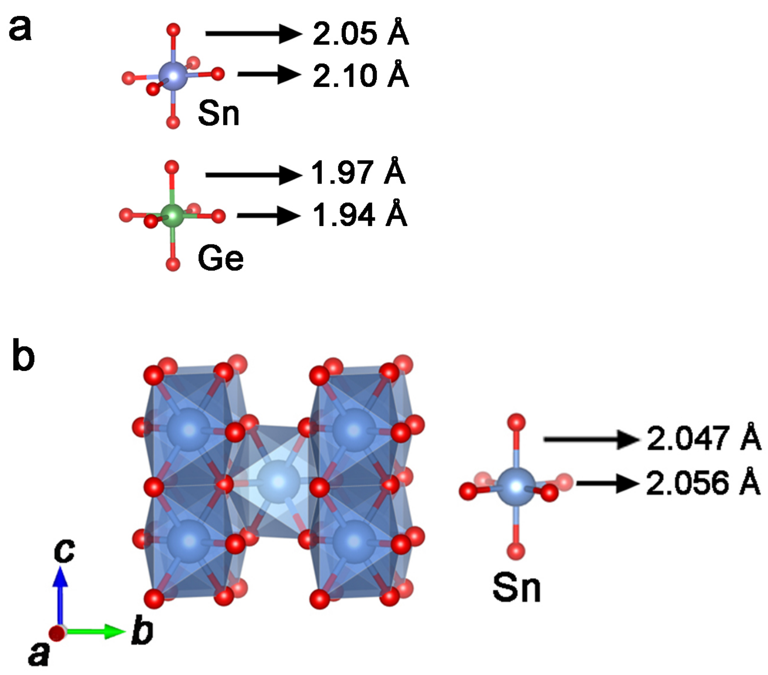

Supplementary Figure 30. (a) The bond length of Sn-O and Ge-O in SGO; (b) The bond length of Sn-O in SO. The red, blue, and green atoms represent O, Sn, and Ge, respectively.

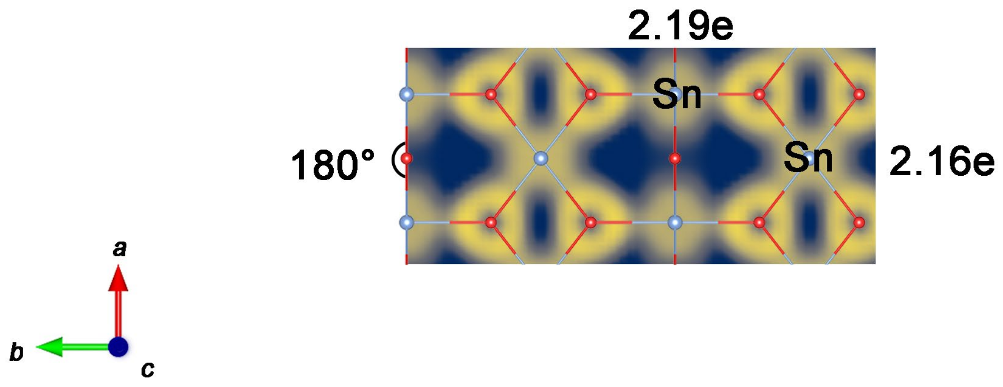

Supplementary Figure 31. The electron local function (ELF) and the Bader charge analysis of SO.

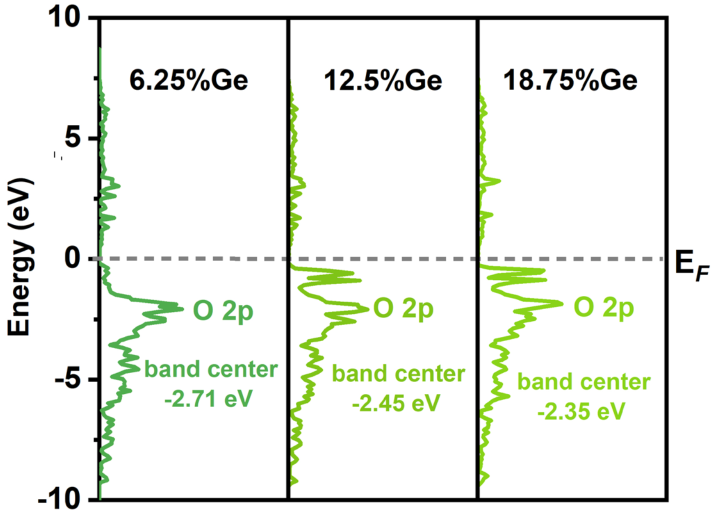

Supplementary Figure 32. The electronic density of states (DOS) of (a) 6.25%Ge-doped SO, (b) 12.5%Ge-doped SO and (c)18.75%Ge-doped SO, respectively. Source data are provided as a Source Data file.

The impact of the germanium ratio on *p*-band center of SO is investigated. we investigated *p*-band centers of SO with Ge doping levels of 0%, 6.25%, 12.5%, 18.75% and 25%, respectively (Supplementary Figure 32). The findings indicate that the *p*-band center of O rises proportionally with the increase in Ge doping amount.

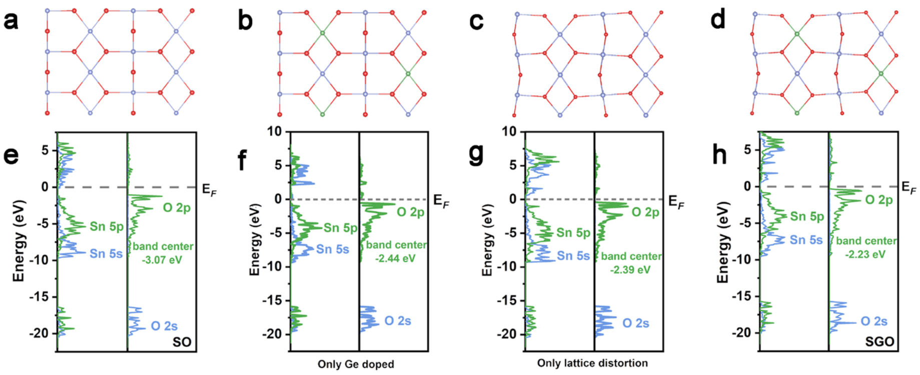

Supplementary Figure 33. Crystal structure and electronic density of states of (a, e) SO, (b, f) Ge doped SO without lattice distortion, (c, g) lattice-distorted SO but without Ge doping and (d, h) normal Ge doped SO. Source data are provided as a Source Data file.

To investigate the impact of lattice distortion and Ge electronic interaction on the *p*-band center of SO, we established four models: 1) a normal SO model (Supplementary Figure 33a), 2) a Ge-SO model with Ge doping but no lattice distortion (Supplementary Figure 33b), 3) a lattice-distorted SO model without Ge doping (Supplementary Figure 33c), and 4) a normal Ge-SO model (Supplementary Figure 33d). The *p*-band centers of these models were calculated from DOS. The results demonstrate that both the doping of Ge and the distorted SO lattice cause an upward shift in the *p*-band center of SO. However, the degree of upward shift is lower when only Ge is doped or only lattice distortion compared to the combination of both factors (Supplementary Figure 33e-h). This confirms that the two changes induced by the doping of Ge collectively modify the electronic structure of SO.

Supplementary Table 1. The crystallographic information for SO and SGO according to the XRD profile fitting results.

Chemical name	Stannic oxide	Germanium-doped Stannic oxide
Chemical formula	SnO_2	$Sn_{0.8}Ge_{0.2}O_2$
Crystal system	Tetragonal	Tetragonal
Space group	P42/mnm	P42/mnm
Cell length *a* (Å)	4.7417	4.7211
Cell length *b* (Å)	4.7417	4.7211
Cell length *c* (Å)	3.1894	3.1697
Cell angle *α* (°)	90.0	90.0
Cell angle *β* (°)	90.0	90.0
Cell angle *γ* (°)	90.0	90.0
Cell size (Å^3)	71.71	70.65

Supplementary Table 2. The peak shift information for SO and SGO according to the XRD patterns. (# means that the diffraction peaks at this position are too broadened to be precisely localized)

Number	2 Theta-SO (°)	2 Theta-SGO (°)	Peak shift (°)
1	26.45	26.72	0.27
2	33.75	34.04	0.29
3	37.82	38.18	0.35
4	38.85	#	-
5	51.70	52.18	0.48
6	54.63	#	-
7	57.79	#	-
8	61.86	62.39	0.53
9	64.66	#	-
10	65.86	#	-

Supplementary Table 3. The hydrogen sensing characters of SGO and previously-reported materials.

Sensing materials	Operating temperature (℃)	Concentration (ppm)	Response (R_a/R_g)	Response/ Recovery time (s)	Reference
CeO_2-SnO_2	300	60	1323	17/24	1
Pd-SnO_2/rGO	360	200	32.4	25.8/31.2	6
SnO_2-D	250	6	2.21	7/12	7
Er-SnO_2	360	100	28	11/42	2
Pd-SnO_2	160	100	28	4/-	4
Pt/SnO_2	200	250	51.6	35/320	9
3D Cu-Doped SnO_2	180	100	45%	18/84	8
p-Sb_2O_3/*n*-SnO_2	280	100	697	29.8/13.4	5
Pd-SnO_2	300	100	56	22/164	3
**Ge-SnO_2**	**220**	**1000**	**63.93**	**2/58**	**This work**

Supplementary Table 4. BET surface areas (BET) of $Sn_{(1-x)}Ge_xO_2$ (x= 0, 0.1, 0.2, 0.3, 0.4) and GeO_2.

Sample	BET (m^2/g)
SnO_2	27
$Sn_{0.9}Ge_{0.1}O_2$	96
$Sn_{0.8}Ge_{0.2}O_2$	113
$Sn_{0.7}Ge_{0.3}O_2$	177
$Sn_{0.6}Ge_{0.4}O_2$	151
GeO_2	0.18

Supplementary Table 5. The possible bulk structures of SGO with germanium content from 6.25% to 25%, and the related total energies in DFT calculations. The model with the lowest total energy in each germanium content (red marked) was chosen as the corresponding SGO bulk model.

Sn: Ge	Sn	Ge	O
1:0	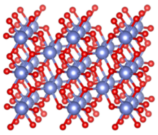<br>E_{total} = -300.52 eV		
15:1 (6.25%)	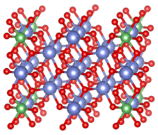<br>E_{total} = -300.52 eV		
14:2 (12.5%)	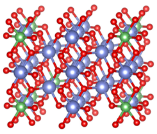<br>E_{total} = -300.56 eV	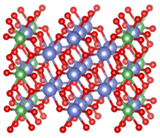<br>E_{total} = -300.38 eV	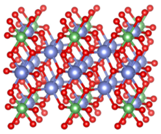<br>E_{total} = -300.67 eV
	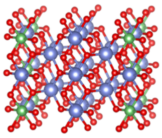<br>E_{total} = -300.62 eV	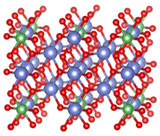<br>E_{total} = -300.42 eV	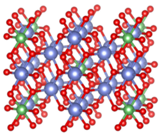<br>E_{total} = -300.50 eV
13:3 (18.75%)	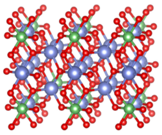<br>E_{total} = -300.75 eV	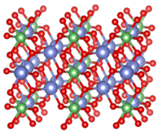<br>E_{total} = -300.64 eV	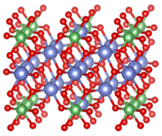<br>E_{total} = -300.74 eV
	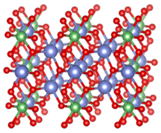<br>E_{total} = -300.76 eV	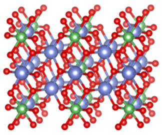<br>E_{total} = -300.18 eV	
12:4 (25%)	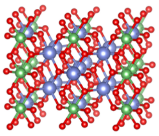<br>E_{total} = -300.88 eV	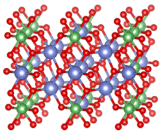<br>E_{total} = -300.68 eV	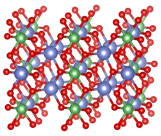<br>E_{total} = -300.63 eV
	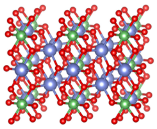<br>E_{total} = -301.01 eV	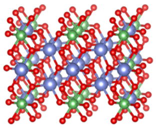<br>E_{total} = -300.94 eV	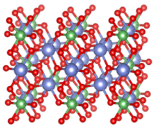<br>E_{total} = -300.89 eV
