# Supplementary material for: Essential role of lattice oxygen in hydrogen sensing reaction
Source: Nat Commun. 2024 Apr 8;15:2998. doi: 10.1038/s41467-024-47078-x (PMC11001979; doi:10.1038/s41467-024-47078-x)
Supplement: Supplementary file 3 — Reporting Summary [file 41467_2024_47078_MOESM3_ESM.pdf]

nature portfolio

Corresponding author(s): Guo-dong Li

Last updated by author(s): Dec 21, 2023

Reporting Summary

Nature Portfolio wishes to improve the reproducibility of the work that we publish. This form provides structure for consistency and transparency in reporting. For further information on Nature Portfolio policies, see our Editorial Policies and the Editorial Policy Checklist.

Statistics

For all statistical analyses, confirm that the following items are present in the figure legend, table legend, main text, or Methods section.

n/a	Confirmed	
☐	☒	The exact sample size (*n*) for each experimental group/condition, given as a discrete number and unit of measurement
☐	☒	A statement on whether measurements were taken from distinct samples or whether the same sample was measured repeatedly
☐	☒	The statistical test(s) used AND whether they are one- or two-sided<br>*Only common tests should be described solely by name; describe more complex techniques in the Methods section.*
☐	☒	A description of all covariates tested
☐	☒	A description of any assumptions or corrections, such as tests of normality and adjustment for multiple comparisons
☐	☒	A full description of the statistical parameters including central tendency (e.g. means) or other basic estimates (e.g. regression coefficient) AND variation (e.g. standard deviation) or associated estimates of uncertainty (e.g. confidence intervals)
☐	☒	For null hypothesis testing, the test statistic (e.g. *F*, *t*, *r*) with confidence intervals, effect sizes, degrees of freedom and *P* value noted<br>*Give P values as exact values whenever suitable.*
☐	☒	For Bayesian analysis, information on the choice of priors and Markov chain Monte Carlo settings
☐	☒	For hierarchical and complex designs, identification of the appropriate level for tests and full reporting of outcomes
☐	☒	Estimates of effect sizes (e.g. Cohen's *d*, Pearson's *r*), indicating how they were calculated

Our web collection on statistics for biologists contains articles on many of the points above.

Software and code

Policy information about availability of computer code

Data collection	no software was used
Data analysis	no software was used

For manuscripts utilizing custom algorithms or software that are central to the research but not yet described in published literature, software must be made available to editors and reviewers. We strongly encourage code deposition in a community repository (e.g. GitHub). See the Nature Portfolio guidelines for submitting code & software for further information.

Data

Policy information about availability of data

All manuscripts must include a data availability statement. This statement should provide the following information, where applicable:

- Accession codes, unique identifiers, or web links for publicly available datasets
- A description of any restrictions on data availability
- For clinical datasets or third party data, please ensure that the statement adheres to our policy

The data that support the findings of this study are available within the article and its Supplementary Information. All other relevant data supporting the findings of this study are available from the corresponding authors upon request.

Research involving human participants, their data, or biological material

Policy information about studies with human participants or human data. See also policy information about sex, gender (identity/presentation), and sexual orientation and race, ethnicity and racism.

Reporting on sex and gender	this information has not been collected
Reporting on race, ethnicity, or other socially relevant groupings	this information has not been collected
Population characteristics	this information has not been collected
Recruitment	this information has not been collected
Ethics oversight	this information has not been collected

Note that full information on the approval of the study protocol must also be provided in the manuscript.

Field-specific reporting

Please select the one below that is the best fit for your research. If you are not sure, read the appropriate sections before making your selection.

☐ Life sciences ☐ Behavioural & social sciences ☒ Ecological, evolutionary & environmental sciences

For a reference copy of the document with all sections, see nature.com/documents/nr-reporting-summary-flat.pdf

Ecological, evolutionary & environmental sciences study design

All studies must disclose on these points even when the disclosure is negative.

Study description	This article is a study on the gas-sensing properties of inorganic metal oxides. All experiments and data replications were completed at the State Key Laboratory of Inorganic Synthesis and Preparative Chemistry, Jilin University.
Research sample	SnO2 and germanium-doped SnO2 synthesized by electrospinning method
Sampling strategy	In this paper, we study the gas-sensing properties of our own synthesized metal oxides, focusing on the effect of the electronic structure of the metal oxides on the gas-sensing properties, and no sampling is involved.
Data collection	Data for this article was collected by Jiayu Li. All experiments and data replications were completed at the State Key Laboratory of Inorganic Synthesis and Preparative Chemistry, Jilin University.
Timing and spatial scale	Data collection for this article was done by Jiayu Li for the period 2022-2023
Data exclusions	no data were excluded from theanalyses
Reproducibility	all attempts to repeat the experiment were successful
Randomization	In this paper, we study the gas-sensing properties of our own synthesized metal oxides, focusing on the effect of the electronic structure of the metal oxides on the gas-sensing properties, and no Randomization is involved.
Blinding	In this paper, we study the gas-sensing properties of our own synthesized metal oxides, focusing on the effect of the electronic structure of the metal oxides on the gas-sensing properties, and no Blinding is involved.

Did the study involve field work? ☐ Yes ☒ No

Reporting for specific materials, systems and methods

We require information from authors about some types of materials, experimental systems and methods used in many studies. Here, indicate whether each material, system or method listed is relevant to your study. If you are not sure if a list item applies to your research, read the appropriate section before selecting a response.

Materials & experimental systems

n/a	Involved in the study
☒	☐ Antibodies
☒	☐ Eukaryotic cell lines
☒	☐ Palaeontology and archaeology
☒	☐ Animals and other organisms
☒	☐ Clinical data
☒	☐ Dual use research of concern
☒	☐ Plants

Methods

n/a	Involved in the study
☒	☐ ChIP-seq
☒	☐ Flow cytometry
☒	☐ MRI-based neuroimaging

Plants

Seed stocks	No plants were involved in this study
Novel plant genotypes	No plants were involved in this study
Authentication	No plants were involved in this study
